# The Transition from Crawling to Walking: Can Infants Elicit an Alteration of Their Parents’ Perception?

**DOI:** 10.3389/fpsyg.2016.00836

**Published:** 2016-06-01

**Authors:** Claudio Longobardi, Rocco Quaglia, Michele Settanni

**Affiliations:** Department of Psychology, University of TurinTurin, Italy

**Keywords:** parents’–infant interaction, developmental transition, crawling, walking, infancy, locomotor, unintentional injury, perception

## Abstract

Our study was designed to address a gap in the literature on parents’ perception and motivation to protect their infants from potential risk of injury in the transition from crawling to walking. The participants were 260 Italian subjects, of whom 158 were women and 102 men, aged between 20 and 45 years. They were asked to draw two domestic objects (a kitchen table and a CD cover) to assess the possible alterations in the perception of environmental elements seen by the parents as a potentially dangerous cause of unintentional injury for their child. Analysis showed that the group of mothers with children aged 9–18 months had drawn the largest tables, while the table areas of the other two categories of women were much smaller. As for the males, the group that drew the largest tables was the one with children, but not in the age range of 9–18 months, while there was little difference between the other two groups. The final descriptive analysis concerned the average scores on the STAI-Y tests both for state and trait anxiety. In all groups a substantial parity was observed, except for the non-parent men, who had a lower level of state anxiety. Both the fathers and the mothers of children aged 9–18 months obtained lower scores, both for state and trait anxiety. Based on the findings, we demonstrate that children transitioning from crawling to walking can elicit a perceptive reactivity in their mothers, which satisfies their natural need to protect their offspring.

## Introduction

Development processes often include a series of qualitative transitions serving to acquire more successful strategies than those already mastered. One of the most significant examples of this process of change and qualitative development is the change from crawling to walking (Adolph and Tamis-LeMonda, 2014). This transition in fact involves a new coordination of the limbs, keeping an upright posture and a new equilibrium of the body with the environment. Although the process of change can last months (Hallemans et al., 2006), some studies have showed the costs and benefits of this transition. For example, Adolph and Tamis-LeMonda (2014) suggest that novice walkers insist on walking instead of crawling because they can go further faster, the erect position gives greater visibility to the area to be explored (Kretch et al., 2014) and infants can interact with objects in a qualitatively different way (Barasik et al., 2013).

### Parental Interaction during the Crawling-to-Walking Transition

The development of independent mobility changes the interaction of infants with their primary caregivers (Biringen et al., 2008). The transition from crawler to novice walker is illustrated by the concept of Developmental Cascade: children increase the interaction with their mothers (Clearfield, 2011) since they have their hands free to be able to share objects in a qualitatively different way, while keeping their attention on the object (Karasik et al., 2011; Yu and Smith, 2013). This change accompanying the transition therefore offers new opportunities to share objects. Furthermore, the child’s transition to walking has been found to be associated with a change in maternal behavior, especially at the verbal level (Karasik et al., 2014). Various studies have analyzed the verbal responses to the children’s bids, or object sharing. For instance, mothers respond to infant bids more frequently than to exploration or play (Bornstein et al., 2008). They are also more likely to label the referent of a developmentally advanced gesture like showing an object than in cases of gestures emerging earlier like pointing or requesting (Olson and Masur, 2011) and the main words used to ward off possible dangers are “No! Don’t! Stop!” (Tamis-LeMonda et al., 2007).

### Children’s Risk of Injury and its Influence on Parental Behavior and Perception

Developmental literature has highlighted the importance of early influence in protecting and promoting healthy development. The infant–caregiver relationship has been widely recognized to play an important role in child development in particular in the transition from crawling to walking (Adolph and Tamis-LeMonda, 2014). Since perception systems have not matured fully at birth, the biological process of maturing along with experience further refines perception, which allows infants to explore their surroundings (Kretch et al., 2014). There is ample evidence that experience is important for the perception of affordances (Adolph et al., 1993). Perceptual attunement refers to perceptual changes over a period of practice with the informational variables upon which actions rely (Fajen et al., 2009). Various studies indicate that infants perceive what the environment offers and suggest that the perception of danger comes from an active exploration of the world (Schwebel et al., 2009).

However, the relation between body and environment is not easily perceived by infants during early development. Until their second year of life, infants often make mistakes of self-awareness that are connected to the size of their body (e.g., trying to go through doors that are too narrow), or related to the inability to perceive their body as an obstacle (e.g., trying to push a stroller attached to a blanket where they are standing without realizing that they have to remove themselves from the blanket; Brownell et al., 2007). Such errors could in fact lead to accidents and injuries. There is support for the hypothesis that the probability of accidents increases in periods of rapid bodily change and in the early phase of acquiring motor skills (van Hof, 2005; Schwebel et al., 2009). Although the transition is advantageous in developmental terms, it exposes children to potential dangers. In fact, infants can fall and hit their heads, swallow dangerous liquids, cut themselves with glass, get run over or burnt. It is well documented that unintentional injuries are the main cause of injury and death in children aged between 1 and 4 years (Hoyert et al., 2006).

To our knowledge a few epidemiological researches have investigated the changed risk of injuries in young children according to developmental phase. However some authors (MacInnes and Stone, 2008) published data indicating accidents during crawling and/or walking as a more frequent cause of injury in the age group 12–35 months (18.7%) than in the age group 0–11 (10.0 %).

According to the World Report on Child Injury Prevention (Peden et al., 2008) every day all over the world over 2000 families lose a child due to unintentional injury.

Given the dangers for infants during this phase of development of independent locomotion, parents are concerned and anxious about their children’s safety and are ready to implement numerous strategies designed to guarantee safety, such as modeling or organizing the environment according to their child’s developmental stage, or removing objects that are considered dangerous (Morrongiello, 2005; Cordovil et al., 2015).

Numerous studies (Plumert, 1995; Phelps et al., 2006; Clearfield et al., 2008; Kretch et al., 2014) have also demonstrated that during the crawling-to-walking transition, as in the non-locomotor to locomotor transition, parents’ behavioral changes are accompanied by changes in their inner states in relation to motivation, the priority of life goals, and feelings connected to their relationship with the infant. Past studies (see Olson and Masur, 2011; Zadra and Clore, 2011) have demonstrated that affective valence and arousal convey information about the importance and value of objects and events, and that such information is embedded in the visual perception of the environment. For instance, fear increases the chances of seeing potential threats and tends to change the way of seeing things. Research shows that the objects in the environment which are emotionally and motivationally relevant attract attention and can be more easily detected by appearing larger (Campos et al., 1992; Adolph and Avolio, 2000; Stefanucci and Storbeck, 2009). Indeed, there is evidence that perception is systematically altered in ways that can help achieve objectives and that emotion can alter spatial perception to motivate choices of economic action and discourage potentially dangerous actions. Affective information provokes immediate and automatic effects on perception without reflection on the significance and sense of emotionally evocative stimuli or the consequences of potential actions (Storbeck and Clore, 2008).

According to the literature, it can therefore be hypothesized that changes in parents’ inner states may be reflected in alterations in their perception of the environment and especially of potentially dangerous objects (Clearfield et al., 2008; Stefanucci and Storbeck, 2009). More specifically, we expect that the objects that put the child’s safety at risk may become emotionally and motivationally important for the parent, arousing a negative emotional reaction, and that they may therefore be associated with a systematic alteration of the parent’s perception, leading in particular to an increase in the perceived size of the dangerous objects.

Some studies, however, reveal behavioral differences between mothers and fathers. Mothers tend to adopt parental choices that are more oriented to safety, while fathers tend to support challenges (Ishak et al., 2007). In addition, the parents’ anxiety level may be a factor influencing the mode of protecting and caring for children during this phase, and this influence may be reflected in the way the environment is perceived (Storbeck and Clore, 2008).

Although various studies (e.g., Stuchlìkovà, 1995; Fox et al., 2001) showed that anxious individuals interpret objects as being more threatening compared to non-anxious individuals, no research has dealt with these phenomena of interaction between caregiver and child during the transition from crawling to walking. Although there are studies (Orbán and Dastur, 2012; Roos and Lochner, 2012), that show a distortion of perception in pregnant mothers, especially in relation to stimuli that might be important for the survival or safety of the fetus, to our knowledge no studies have yet dealt with verifying the presence of perceptive alteration in the parent during this transition.

### Current Study

Our study was designed to address a gap in the literature on the influences of emotional and motivational factors on parents’ visual spatial perception of objects potentially dangerous for their children.

We hypothesize that changes in visual spatial perception are consistent with an adaptive view of the parental desire to protect infants in the specific, potentially risky, stage of locomotor development. From an evolutionary point of view, even a small increase in the ability to detect potential environmental dangers should give the infant a survival advantage during this transition. In this phase, infants will stimulate maternal reactivity with their behavior, not only on a verbal level, as argued by Karasik et al. (2014), but also on the level of perception. In our view, this occurs through the special parent–infant dyadic interaction, which affect the parents’ internal state, which in turn motivates them to protect their children.

The goal of the present study is to examine whether or not there is a change in parents’ perception of environmental elements that could threaten the infant’s safety. Specifically, we test two hypotheses: (a) parents with infants aged 9–18 months which have been walking for less than 4 months will exhibit a different perception of the potential risk of environmental elements when compared with parents with infants in other age ranges. Our prediction is that, when asked to draw the kitchen table in a kitchen, parents with infants in the 9–18-month-age range will draw significantly larger tables; (b) no changes in the spatial perception of non-potentially harmful elements will emerge. Our prediction is, when asked to draw an object that is not potentially harmful (e.g., a compact-disk cover), the areas of the drawings made by parents with children in the 9–18-month-age range will not differ significantly from the ones drawn by other participants.

## Materials and Methods

### Participants

The sample was made up of 260 subjects of Italian nationality (158 females and 102 males), aged between 20 and 45 years. The average age for males was 35.4 years (*SD* = 6.4), and 33.1 years (*SD* = 5.8) for females.

The subjects, both male and female, were divided into three sub-groups: Non-Parent (*n* = 94, females = 60%); Parent with child of 9–18 months which has been walking for less than 4 months (*n* = 79; females = 63%; children’s age: *M* = 13.3 months, *SD* = 3.1); Parent with child aged over 18 months which has been walking for at least 4 months (*n* = 87; females = 60%; children’s age: *M* = 41.6 months, *SD* = 24.3).

### Instruments

#### VOSP – Visual Object and Space Perception Battery (Warrington and James, 1991)

The battery can be divided into two parts: the first part assesses the possible presence of an impairment in spatial perception; the second part identifies possible problems in the visual identification of objects. The subjects participating in this research were tested solely by measuring their spatial perception, so as to exclude participants with a low level in these skills. We used the following sub-tests: dot counting, position discrimination, and number location.

#### STAI-Y – State–Trait Anxiety Inventory (Form Y; Spielberger et al., 1983)

STAI-Y is an inventory that can be self-administered, which reveals and measures a subject’s level of anxiety. The inventory is made up of 40 items, to which subjects answer in terms of intensity (i.e., from “hardly ever” to “nearly always”). The items are subdivided into two scales: State Anxiety, which is considered an experience of insecurity, impotence or particular concern, at the time when the questionnaire is compiled, and Trait Anxiety, which is the stable tendency to perceive stressful situations as dangerous and threatening and to react to different situations with more intense degrees of anxiety. Each item is assigned a score from 1 to 4 and the total score on each scale ranges from 20 to 40. The full version of the test has quite high reliability both for State (Cronbach’s Alpha = 0.91), and for Trait anxiety (Cronbach’s Alpha = 0.90).

### Experimental and Control Tables

In order to test our hypothesis, we developed two different drawing tasks. The participants were asked to draw specific objects recognized as potentially dangerous or non-dangerous for children during the crawling-to-walking transition. The choice of the objects to be drawn was based on the results of two focus groups conducted with parents of children in the 9–18-month-age range (*N* = 16). A discussion was held with these parents on the dangers to which children are exposed in the home. Various objects or furnishings were mentioned by the parents as being dangerous for children learning to walk. Those considered most dangerous were stairs, followed by table, chair, highchair, and lounge room furniture. Of these, we chose the kitchen table because it is found in all houses, and is regarded as one of the greatest potential sources of unintentional injury, since children run the risk of bumping into it or crashing into its corners, for example. The kitchen is also one of the rooms where parents and children spend the most time together. The second object that was selected was a CD cover, because the focus groups almost unanimously recognized CD cases as non-dangerous objects which are present in every household.

**FIGURE 1:**
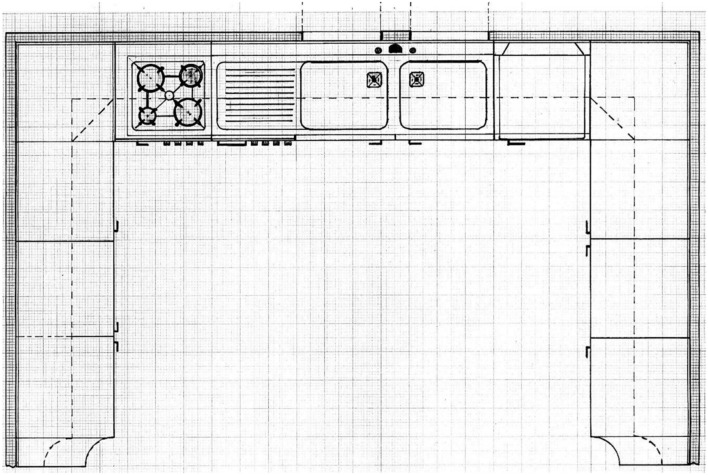
**Experimental table**.

For the first drawing task, the subjects were asked to draw a table inside a kitchen plan provided by the researchers in the format of an A4 sheet (see **Figure 1**). We used graph paper so as to have measurements that were as accurate as possible, which could be used to analyze the possible differences between groups.

**FIGURE 2:**
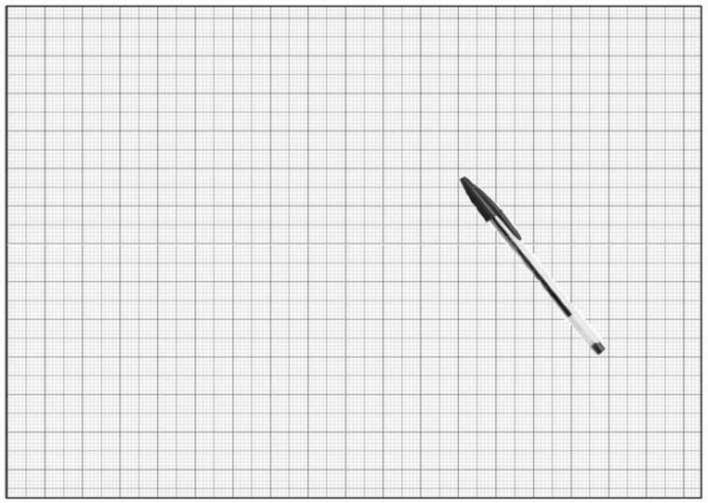
**Control table**.

The control table consisted of the surface of a table as seen from above, placed in the center of an A4-sized sheet (see **Figure 2**): the subjects were asked to draw a CD cover and were given no additional instructions. Graph paper was also used in this case, in order to get exact measurements. A ballpoint pen was printed on the sheet, in order to give the participants a spatial reference point concerning the overall distance and size of the table. The underlying principle of this test was that, since the CD cover would not be considered dangerous for the child’s safety during the transition from crawling to walking, there should not be any significant differences in the way it was drawn by all the groups of individuals in our sample.

### Compliance with Ethical Standards

Individual informed consent to take part in the research was collected from participants, along with written consent describing the nature and objective of the study according to the ethical code of the Italian Association for Psychology (AIP) and with adherence to the privacy requirements required by Italian law (Law DL-196/2003). As regards the ethical standards for research, the study complied with the latest version of the Declaration of Helsinki (World Medical Association (WMA), 2013).

### Data Analysis

We checked the scores obtained by the participants in the VOSP subtests in order to exclude from the subsequent analyses the individuals who presented problems in spatial perception (none were excluded). We conducted descriptive analyses on the remaining participants: the mean and standard deviations of the variables were calculated: age – adult or child, area of table and CD cover, STAI-Y score. The frequency of male/female children in the groups was recorded and then linked to the ability to walk independently, as well as the number of places at the tables. Subsequently, we conducted inferential analyses. As a preliminary step to test our hypotheses, we compared the groups based on the ‘type of parent’ variable. More specifically, our aim was to test the presence of significant differences concerning potential disturbance variables, that is, the number of places at the participants’ table (having larger or smaller tables at home could influence the size of the drawings), and the two anxiety measures (i.e., State and Trait anxiety). Next, we conducted two factorial ANOVAs, respectively, on the female and male subsamples, testing for the effects of parental type (non-parent vs. parent w/9–18 months child vs. parent w/18+ months) and drawn object (table vs. CD cover) on the surface areas of the drawings, including state- and trait-anxiety in the models.

## Results

### Spatial Perception Analyses

The first analyses carried out were those related to the VOSP test. The VOSP scores were used as an exclusion criterion to remove participants with spatial perception deficits from the sample (we used the cutoff scores proposed by the VOSP manual, which are 8 for Dot counting, 18 for Position discrimination, and 7 for Number location). The scores of all the research participants on the different VOSP subscales were above the deficit threshold, indicating the absence of spatial perception deficit.

### Descriptive Statistics

**Table 1** presents descriptive statistics of the research participants. The average age of the participants differs significantly across the three groups. Both male and female non-parents are significantly younger than the parents, and mothers of children aged 9–18 months are significantly younger than mothers with children aged over 18 months [males: *F*(2,99) = 10.79, *p* < 0.001; females: *F*(2,155) = 9.29, *p* < 0.001].

**Table 1:** Means and standard deviations of age of participants.

	Groups	N°	Means age	*SD*
Male	Non-parent	38	31.9	6.7
	Parent with child of 9–18 months	29	37.1	4.9
	Parent with child over 18 months	35	37.8	5.5
	**Total**	102	35.4	6.4

Female	Non-parent	56	30.9	6.4
	Parent with child of 9–18 months	50	33.2	4.7
	Parent with child over 18 months	52	35.5	5.3
	**Total**	158	33.1	5.8


**Table 2** shows the statistics describing the area of the objects drawn by the participants (i.e., the kitchen table and CD cover). Once the dependent variables of the research were analyzed, namely the areas of the drawings of the kitchen table and CD cover, it was seen that the group of mothers with children aged 9–18 months had drawn the largest tables, while the table areas of the other two female categories were much smaller.

**Table 2:** Descriptive statistics of table area and CD case area.

	Groups	Table area		CD case area	
			
		*M*	*SD*	*M*	*SD*
Male	Non-parent	4051.11	1634.87	2787.87	1736.32
	Parent with child of 9–18 months	3372.93	1767.56	2465.93	2692.64
	Parent with child over 18 months	4296.43	1908.87	2397.40	1736.78
Total male		3942.47	3942.47	1792.45	2562.35

Female	Non-parent	3464.13	1767.05	2642.68	1505.12
	Parent with child of 9–18 months	4635.96	2046.46	2707.46	2764.54
	Parent with child over 18 months	3602.13	1821.07	2304.63	1658.64
Total female		3880.38	1935.84	2551.92	2025.70

Total participants		3904.74	1877.62	2556.02	2027.34


As regards males, the group that drew the largest tables was the one with children, but not in the age range of 9–18 months, while there was little difference between the other two subgroups.

The final descriptive analysis concerned the average scores on the STAI-Y tests both for state and trait anxiety (see **Table 3**). A substantial parity was observed in all groups, except for the non-parent males, who exhibited a lower level of State anxiety. Both the fathers and the mothers of children aged 9–18 months obtained lower scores for State and Trait anxiety.

**Table 3:** Means and standard deviation of STAI-Y State and Trait.

	Groups	STAI-Y (State)		STAI-Y (Trait)	
			
		*M*	*SD*	*M*	*SD*
Man	Non-parent	2.07	0.38	2.20	0.32
	Parent with child of 9–18 months	2.13	0.46	2.10	0.26
	Parent with child over 18 months	2.19	0.27	2.15	0.18
Total man		2,13	0.37	2.15	0.26

Woman	Non-parent	2.02	0.43	2.11	0.38
	Parent with child of 9–18 months	1.96	0.44	2.04	0.34
	Parent with child NOT of 9–18 months	2.12	0.35	2.08	0.29
Total woman		2.03	0.41	2.08	0.34

Total participants		2.07	0.40	2.10	0.32


### Inferential Analysis

In order to test for significant differences in the potential disturbance variables levels between the groups defined by parental type, we conducted ANOVAs setting the number of places, STAY-T score, and STAY-S score as DV, and setting the parent categories (non-parents, parents with children of 9–18 months and parents with children over 18 months) as the IV. The analyses were conducted separately for males and females. No significant effects emerged from the analyses [Males: number of places: *F*(2,99) = 0.47, *p* = 0.63; STAY-T: *F*(2,99) = 1.27, *p* = 0.29; STAY-S: *F*(2,99) = 0.88, *p* = 0.42; Females: number of places: *F*(2,155) = 0.19, *p* = 0.98; STAY-T: *F*(2,155) = 0.45, *p* = 0.64; STAY-S: *F*(2,155) = 2.04, *p* = 0.13].

Next, we conducted two separate factorial ANOVAs on the male and female subsamples to identify possible differences in the surface area of both the table and the CD cover among the groups defined by parental type, including age, state- and trait-anxiety variables as covariates in the models. Concerning the size of the table area, a significant interaction between drawn object and parental type emerged [*F*(2,149) = 3.961, *p* = 0.021] in the female group. However, no significant differences emerged in the male subsample [*F*(2,93) = 0.509, *p* = 0.60]. No significant effects emerged regarding the interaction term between parent anxiety (both state and trait) and drawn objects.

*Post hoc* comparisons using the Bonferroni test indicated that the mean areas of the table drawn by women with children aged 9–18 months were larger (*M* = 4635.96, *SD* = 2046.46) than those drawn by both women with no children (*M* = 3464.13, *SD* = 1767.05) and by women with children of different ages (*M* = 3602.13, *SD* = 1821.07). Furthermore, the mean area of the table drawn by women with no children did not significantly differ from the table area drawn by women with children of different ages.

## Discussion

The transition from crawling to walking is a very important phase in children’s development, during which they experience major qualitative changes, such as the acquisition of a new visual vantage point (Kretch et al., 2014), which gives them a reason to interact in a different manner with parents and objects. Children also experience changes in the way they explore the environment, which at the same time expose them to possible safety risks (Peden et al., 2008). Children’s new exploratory behavior elicits new reactions from their parents (Tamis-LeMonda et al., 2007), who play a crucial role in facilitating or hindering the process of transition from crawling to upright locomotion (Brussoni and Olsen, 2013). With this study, we wanted to share the discovery of an alteration in the mother’s perception of potentially dangerous objects present in the child’s environment.

Studies on visual and spatial alteration in perceiving features of the environment have already been conducted (Zadra and Clore, 2011), including the alteration of the perception of objects. For instance, desirable objects are perceived as being closer than non-desirable ones, or threatening objects are seen as being larger than neutral or positive ones (van Ulzen et al., 2008). However, no study has considered the possibility that children who are transitioning from crawling to walking can elicit perceptive reactivity in their mothers, which satisfies their need to protect their offspring.

Our results, which show a major difference between the areas of the tables drawn by the different groups in which our sample was divided (5814.75 cm^3^ in the 9–18-month-old group; 4707.31 cm^3^ in the group of parents with older children), indicates a distortion in the perception of the table by the mothers of children transitioning from crawling to walking. In fact, the table is drawn larger by mothers of children in this transition phase, and this could be considered as indirect evidence of the influence on perception of objects which are emotionally and motivationally relevant. Indeed, given its potential danger, we suppose that the table attracts the attention of mothers, who are motivated to protect their child from risks. This could cause the alteration in perception (the table appears larger) and might serve to protect the child from potential dangers.

The altered perception of the size of the table involved only mothers and not fathers, further reinforcing the data in the literature on gender differences: mothers tend to make protective parental choices that are more oriented to safety, while fathers tend to support challenges, manifesting less hyper-protective behavior (Ishak et al., 2007). As suggested by Morrongiello and Dawber (2000), this can be explained by the fact that fathers believe that unintentional injuries are a natural result of the development process. Therefore, the components of the children’s environment are perceived as being more threatening by mothers than by fathers.

The explanation of the distortion described so far is also supported by the results of the CD cover control table drawing, which shows no difference in all groups, with no distinction between males and females, the means being very similar in both cases. The absence of a distortion in perception could be due to the fact that as the CD cover is not considered dangerous for the child, especially in relation to the transition from crawling to walking, there are no emotional tensions that can affect perception.

Furthermore, we wanted to investigate whether altered perception in mothers during this delicate transition phase could be associated with State or Trait anxiety. In fact, we expected the more anxious and hyper-protective mothers to draw the table larger than it was in reality. The lack of a significant relation between perception and anxiety further supports the independence of the two factors.

## Conclusion

In this study, we have demonstrated a distorted perception that is found in mothers of children aged 9–18 months, while no similar alteration was found in fathers. Mothers, “see the world through their children’s eyes,” with an altered perception of the objects considered potentially dangerous in the environment the infant is exploring. In other words, objects are perceived as being larger than their real size. This alteration is not present in parents of children who have already achieved adequate walking skills, due to the lower level of risk posed to children by objects in the domestic environment.

## Author Contributions

RQ was involved with the design and interpretation of this work as well as writing and revising the manuscript. CL and MS were involved in the acquisition and analysis of the data and contributed to the revising of the manuscript.

## Conflict of Interest Statement

The authors declare that the research was conducted in the absence of any commercial or financial relationships that could be construed as a potential conflict of interest.
